# Cardiac plasmacytoma with extensive infiltration: multimodal diagnosis and therapeutic challenges: a case report

**DOI:** 10.1093/ehjcr/ytag102

**Published:** 2026-02-06

**Authors:** Pastora Rodríguez-Fraga, Enric Cascos, Adriana Cuartas, Ignasi Espadaler

**Affiliations:** Cardiology Department, Hospital Clinic Barcelona, Villaroel 170, 08036 Barcelona, Spain; Cardiology Department, Hospital Clinic Barcelona, Villaroel 170, 08036 Barcelona, Spain; Hemathology Department, Hospital Clinic Barcelona, Villaroel 170, 08036 Barcelona, Spain; Pathology Department, Hospital de la Santa Creu i Sant Pau, Sant Quintí 89, 08041 Barcelona, Spain

**Keywords:** Cardiac plasmacytoma, Cardio-oncology, Cardiac magnetic resonance, Cardiac computed tomography, Echocardiography, Positron emission tomography-computed tomography, Case report

## Abstract

**Background:**

Cardiac involvement in multiple myeloma (MM) in the form of cardiac plasmacytoma is exceedingly rare and carries a poor prognosis.

**Case summary:**

We describe the case of a 59-year-old man with serological relapse of MM in whom a hypermetabolic cardiac mass was detected on positron emission tomography–computed tomography. Cardiac magnetic resonance imaging revealed extensive atrial infiltration with imaging features suggestive of malignancy. Histopathological examination confirmed the diagnosis of cardiac plasmacytoma. The patient was started on systemic anti-CD38 based therapy, which led to a rapid and substantial reduction in tumour burden. Persistent metabolic activity prompted the addition of localized radiotherapy and treatment escalation with a novel bispecific antibody.

**Discussion:**

This case contributes to the limited literature on cardiac plasmacytoma. The role of multimodality imaging for mass characterization is highlighted. Yet, imaging characteristics alone may be indistinguishable from those of primary cardiac malignancies, making tissue diagnosis crucial. Despite advances in systemic therapies that may induce marked tumour responses, long-term prognosis remains guarded. Notably, this case represents one of the longest reported survivals among patients with cardiac involvement.

Learning points

- Cardiac plasmacytoma is a rare manifestation of extramedullary disease in multiple myeloma and is associated with poor prognosis.
- Multimodal imaging and histological confirmation are essential for accurate diagnosis.
- Novel therapeutic agents, including bispecific antibodies, may induce significant tumour regression, but durable remission remains uncommon.

## Introduction

Multiple myeloma (MM) is a clonal plasma cell malignancy commonly confined to the bone marrow and skeleton, defined by the classic CRAB features: hyperCalcaemia, Renal insufficiency, Anaemia, and Bone lesions. A subset of patients develops extramedullary disease (EMD), characterized by soft tissue masses known as plasmacytomas, with an incidence of up to 14% over the course of the disease.^1^ The most frequently affected sites include the skin, muscle, pleura, lymph nodes, liver, and central nervous system, although any organ can be involved.^2^ Extramedullary disease is associated with a more aggressive clinical course, with shorter progression-free and overall survival.^3^

Cardiac involvement by plasmacytoma is exceptionally rare and poses major diagnostic and therapeutic challenges. Diagnosis relies on the integration of multimodal imaging techniques and histological confirmation. Owing to the rarity of this manifestation, evidence-based therapeutic strategies are lacking, and clinical outcomes are generally poor.

We report the case of a patient with relapsed MM who presented with a large cardiac mass, ultimately diagnosed as plasmacytoma.

## Summary figure

**Figure:**
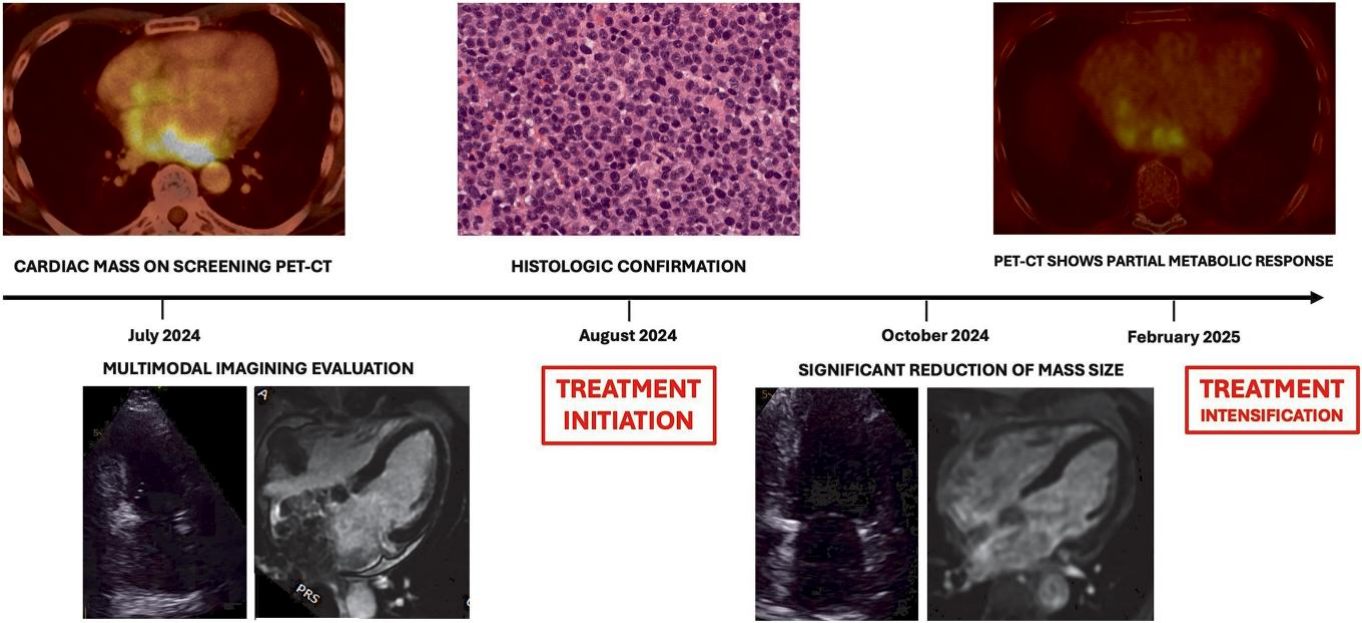


## Case presentation

**Figure 1:**
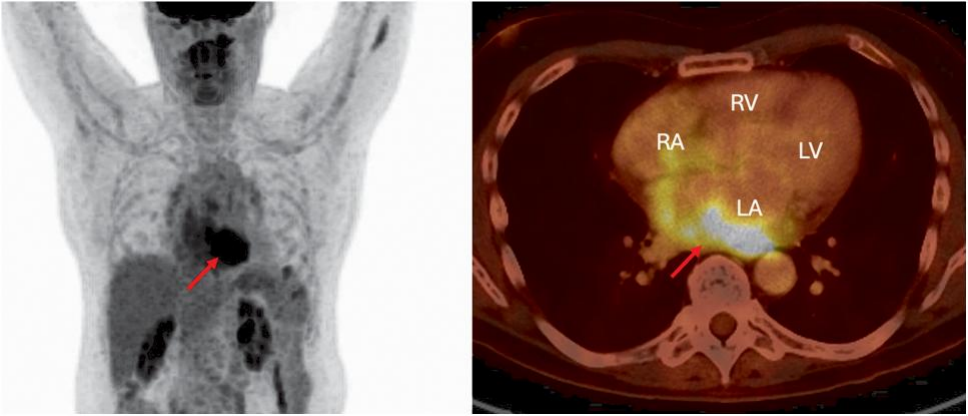
Positron emission tomography–computed tomography reveals a hypermetabolic cardiac mass (arrow) extending from the posterobasal wall of the left atrium to the ostium of the right pulmonary veins.

A 59-year-old man with a history of MM in serological relapse was found to have a large, hypermetabolic cardiac mass on positron emission tomography–computed tomography (PET–CT), performed as part of the work-up for third-line therapy (*Figure 1*). The patient was initially diagnosed with MM in 2019 and received first-line induction chemotherapy with five cycles of bortezomib, thalidomide, and dexamethasone (VTD), followed by consolidation with autologous haematopoietic stem cell transplantation. In 2021, he experienced disease relapse and was treated with chimeric antigen receptor (CAR) T-cell therapy. After a sustained complete response lasting 2.6 years, serological relapse was confirmed in July 2024, and he was considered for third-line treatment with a B-cell receptor bispecific antibody. The PET–CT scan was requested as part of the evaluation process.

Clinically, detection of the mass coincided with patient-reported symptoms of palpitations and exertional dyspnoea. Electrocardiography revealed new-onset paroxysmal atrial fibrillation (AF), with a CHA_2_DS_2_-VA score of 0. No other cardiovascular symptoms were present.

Given the imaging findings on PET–CT, further cardiology evaluation was warranted.

**Figure 2:**
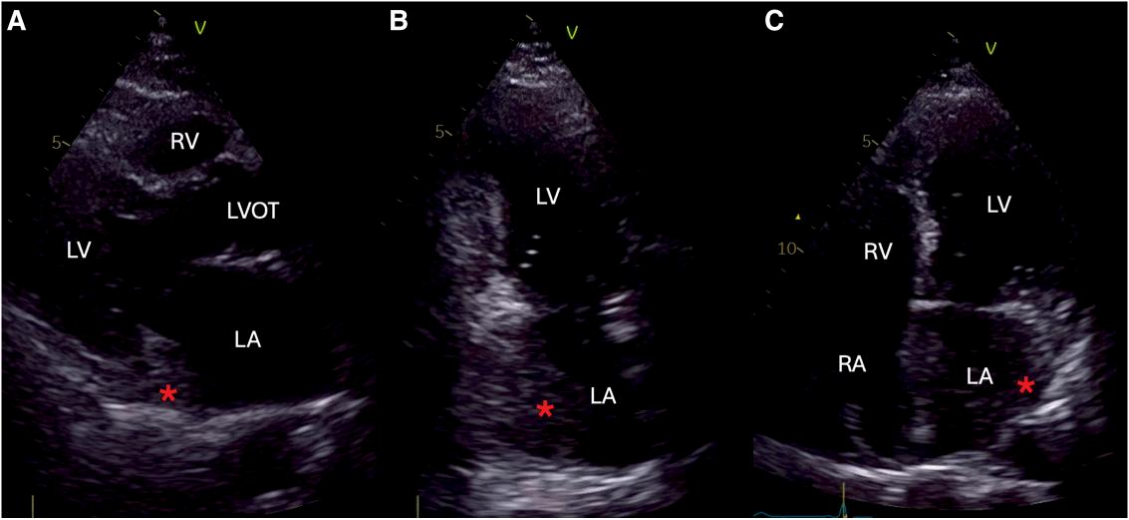
Transthoracic echocardiography shows a hypoechogenic mass (asterisk) occupying the posteroinferior wall of the left atrium, without pedunculation or mobile components. (*A*) Parasternal long-axis view. (*B*) Apical two-chamber view. (*C*) Apical four-chamber view.

Transthoracic echocardiography (TTE) revealed a non-pedunculated mass occupying the roof of the left atrium, with no mobile components on its surface or signs of obstruction (*Figure 2*). The left ventricle was non-hypertrophic, and biventricular systolic function was preserved. Atrial chambers were of normal size, and no valvular abnormalities were present.

**Figure 3:**
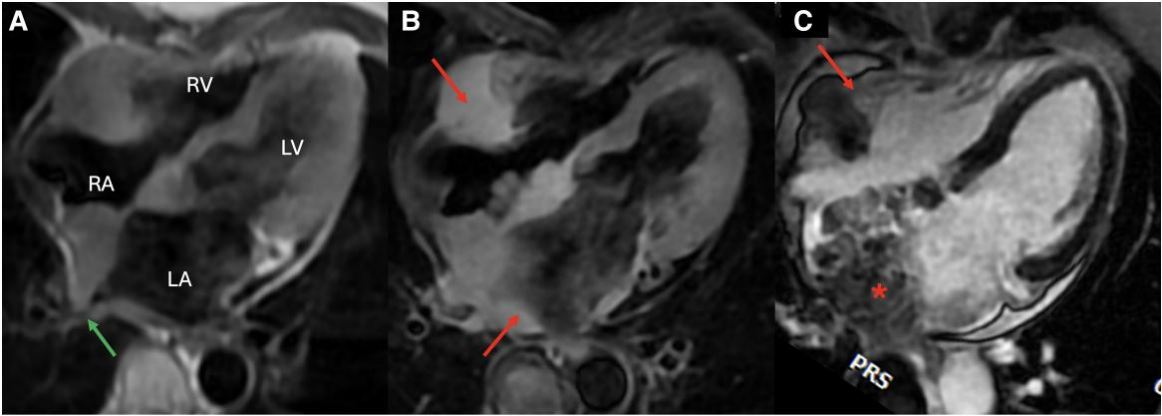
Cardiac magnetic resonance depicts a large cardiac mass measuring 11 × 6.3 cm, with irregular borders and extensive infiltration of both atria and adjacent structures (arrows in B and C). Invasion of the right pulmonary vein ostium is noted, with associated narrowing of the right inferior pulmonary vein (arrow in A). On tissue characterization, the mass exhibits features suggestive of malignancy: (*A*) T1-weighted imaging: the mass is isointense and shows no signal suppression on fat-saturated sequences. (*B*) T2-weighted imaging: the mass is hyperintense, consistent with increased water content and oedema. (*C*) Late gadolinium-enhanced imaging: heterogeneous contrast uptake is observed, with extensive infiltration of the left atrium (asterisk).

Cardiac magnetic resonance (CMR) was performed for further characterization (*Figure 3*). It revealed a large mass (11 × 6.3 cm) extensively infiltrating both atria and adjacent structures. The mass originated in the pre-aortic area, extended along the right atrioventricular groove, and protruded into the right atrium and inferior vena cava. It infiltrated the left atrium and the ostia of the right pulmonary veins, with narrowing of the inferior branch.

On tissue characterization, the mass appeared isointense on T1-weighted sequences, hyperintense on T2-weighted sequences, and showed no suppression on fat-saturated sequences. It demonstrated heterogeneous enhancement on perfusion imaging (see Supplementary material online, Video S1), as well as on early and late gadolinium-enhanced phases. These features were highly suggestive of malignancy. Given the history of MM and the absence of other active lesions on PET–CT, a cardiac plasmacytoma was suspected. However, due to the extensive infiltrative pattern and heterogeneous enhancement, angiosarcoma—the most common primary malignant tumour of the heart—remained an important differential diagnosis.

**Figure 4:**
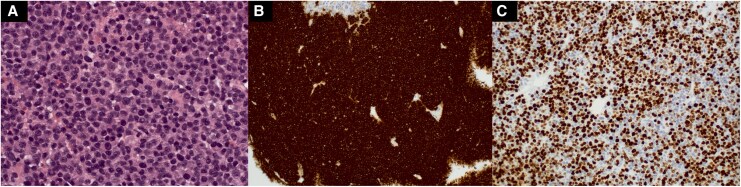
Histopathology. (*A*) Dense neoplastic proliferation of atypical plasma cells. (*B*) Strong and diffuse immunohistochemical staining for CD138, confirming plasma cell lineage. (*C*) High proliferative index, with Ki-67 expression in ∼80% of tumour cells.

A cardiac biopsy was performed. Histopathological examination showed dense myocardial infiltration by atypical plasmacytic cells with evidence of active proliferation (*Figure 4*). Immunohistochemistry confirmed a plasma cell phenotype with diffuse CD138 positivity and lambda light-chain restriction. The proliferation index was high (Ki-67, 80%).

Following multidisciplinary team evaluation, surgical resection was deemed unsuitable due to extensive cardiac infiltration and advanced haematological disease stage. The patient commenced a debulking chemotherapy regimen with anti-CD38 therapy (isatuximab), a proteasome inhibitor (carfilzomib), and dexamethasone. The first two cycles were administered as an inpatient, with close monitoring.

Paroxysmal AF remained the sole complication and was controlled with bisoprolol without haemodynamic compromise.

The patient tolerated therapy well and was safely discharged to continue subsequent cycles in an outpatient setting.

**Figure 5:**
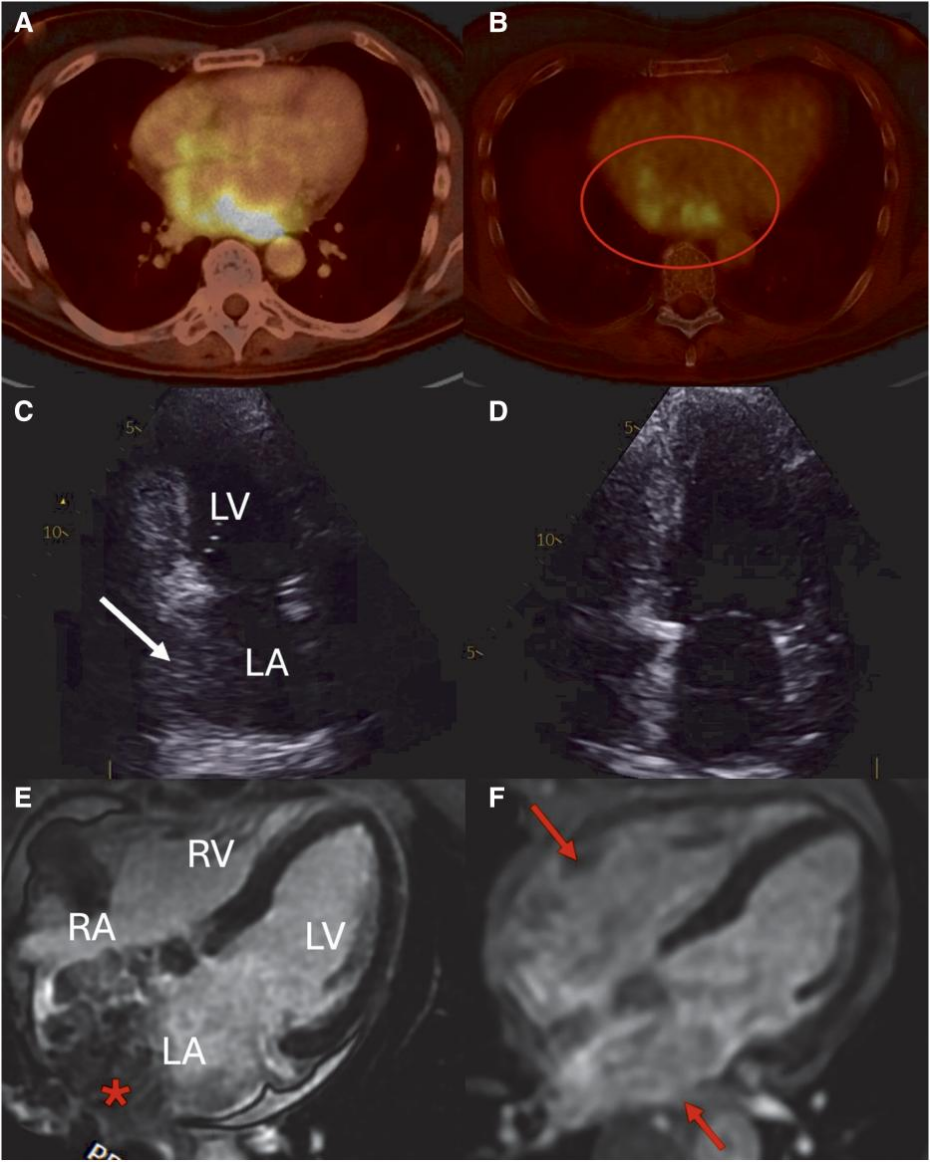
Comparison before and after treatment. Multimodal imaging demonstrates a marked reduction in cardiac mass size following systemic therapy. (*A–C*) Transthoracic echocardiography and cardiac magnetic resonance obtained before treatment show extensive infiltration of both atria and involvement of the right pulmonary vein. Positron emission tomography–computed tomography demonstrates intense fluorine-18 fluorodeoxyglucose (FDG) uptake within the cardiac mass (SUV_max 8), consistent with metabolically active tumour. (*D–F*) Follow-up imaging reveals significant tumour regression. Cardiac magnetic resonance shows residual thickening in the lateral wall of the right atrium (9 mm) and the inferior wall of the left atrium (6 mm). FDG uptake in the cardiac mass (posterobasal aspect of the left atrium) has decreased in both intensity and extent; however, residual metabolic activity persists (SUV_max 6).

At 8-month follow-up, imaging demonstrated a marked reduction in tumour burden. Cardiac magnetic resonance revealed residual thickening of only 9 mm in the lateral wall of the right atrium and 6 mm in the inferior left atrium (*Figure 5*). Clinically, the patient did not experience new episodes of AF. However, PET–CT demonstrated a partial metabolic response and identified a new mediastinal lymphadenopathy.

Owing to persistent metabolic activity and new disease sites, local radiotherapy was administered to the residual cardiac lesion and lymphadenopathy. This was followed by initiation of fourth-line systemic therapy with a novel bispecific antibody targeting B-cell maturation antigen (BCMA).

At ∼17 months from initial presentation, the patient remains alive and continues active treatment.

## Discussion

Cardiac plasmacytoma is an exceptionally rare manifestation of MM and poses significant diagnostic and therapeutic challenges. The incidence of extramedullary disease in MM ranges from 0.5% to 4.8% at diagnosis and from 3.4% to 14% during the course of the disease.^1^ However, only ∼34 cases of cardiac plasmacytoma were reported between 1990 and 2015.^4^

Atrial involvement, particularly of the right atrium, is the most frequently reported site, observed in 42% of cases.^4^ Clinical presentation is often non-specific and varies according to the location and size of the mass. Pericardial effusion occurs in up to 87% of cases, whereas direct myocardial infiltration is less common, reported in only 9%.^4^ In the present case, atrial fibrillation developed secondary to atrial infiltration.

Accurate diagnosis relies on multimodality imaging, with CMR playing a central role in the anatomical and tissue characterization of cardiac masses. Nonetheless, imaging features of cardiac plasmacytoma may closely resemble those of primary cardiac malignancies, as illustrated in this case, in which angiosarcoma was initially considered.

Definitive diagnosis requires histopathological confirmation, obtained via endomyocardial biopsy. This case highlights the importance of pursuing tissue diagnosis when evaluating cardiac masses with malignant imaging features, particularly in patients with known haematological malignancies such as MM.

Extramedullary disease in MM is associated with a more aggressive clinical course and worse overall survival. Reported cases of cardiac plasmacytoma exhibit a particularly poor prognosis, with more than half of patients dying shortly after presentation and only 36% reported alive at the time of publication, with follow-up periods ranging from 1 to 22 months.^4^

The optimal therapeutic approach for cardiac plasmacytoma remains poorly defined. Management options include surgical excision for localized tumours, radiotherapy, and systemic chemotherapy. Emerging immunotherapies, including bispecific antibodies targeting BCMA or other highly expressed plasma cell antigens, have demonstrated efficacy in clinical trials and are approved for the treatment of relapsed or refractory MM.^5^ These agents redirect T-cell cytotoxicity against malignant plasma cells, providing a potential systemic option even in extramedullary disease. However, evidence regarding their efficacy in extramedullary manifestations, particularly cardiac plasmacytoma, remains limited. Further research is required to define their role and guide treatment strategies in this rare and challenging clinical context.

Despite the generally unfavourable prognosis, our patient achieved a rapid and substantial initial response to systemic treatment. Persistent metabolic activity and new nodal involvement, however, necessitated further local and systemic therapy, emphasizing the aggressive nature of the disease. Remarkably, 1 year after diagnosis, the patient remains alive, suggesting a potential benefit from the applied treatment strategy.

## Lead author biography


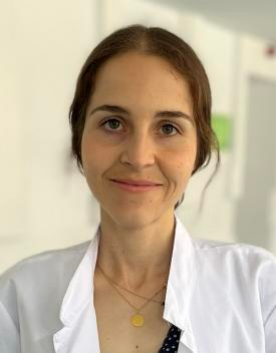


Pastora Rodríguez-Fraga was born in 1997 in Madrid, Spain. She graduated from Complutense University of Madrid in 2021. Currently, she is a fourth-year cardiology resident at the Clinic Hospital of Barcelona, Spain.

## Supplementary Material

ytag102_Supplementary_Data

## Data Availability

The data underlying this article are available in the main manuscript. Due to ethical and privacy concerns, some supporting data cannot be made publicly available but are available from the corresponding authors upon reasonable request.

## Notes

**Consent:** The authors confirm that written consent for the submission and publication of this case report, including images and associated text, has been obtained from the patient in accordance with the COPE guidelines.
